# BrpNAC895 and BrpABI449 coregulate the transcription of the afflux-type cadmium transporter *BrpHMA2* in *Brassica parachinensis*

**DOI:** 10.1093/hr/uhac044

**Published:** 2022-02-19

**Authors:** Shuai Liu, Limei Li, Yanwu Deng, Yongsheng Bai, Chao Sun, Shili Huang, Jiajie Zhou, Liyu Shi, Xuewei Yang, Ling Li, Xuemei Chen, Yulin Tang

**Affiliations:** 1 Guangdong Provincial Key Laboratory for Plant Epigenetics, Shenzhen Key Laboratory of Marine Bioresource & Eco-environmental Science, Longhua Institute of Innovative Biotechnology, College of Life Sciences and Oceanography, Shenzhen University, Shenzhen 518060, Guangdong Province, China; 2 Key Laboratory of Optoelectronic Devices and Systems of Ministry of Education and Guangdong Province, College of Optoelectronic Engineering, Shenzhen University, Shenzhen 518060, China; 3 Shaanxi Academy of Traditional Chinese Medicine, Xi'an, Shaanxi 710003, China; 4 Guangdong Provincial Key Laboratory of Biotechnology for Plant Development, School of Life Sciences, South China Normal University, Guangzhou 510631, China; 5 Life Sciences College, Zhaoqing University, Zhaoqing, 526061, China; 6 College of Horticulture, Nanjing Agricultural University, No. 1 Weigang, 8210095 Nanjing, China; 7 Department of Botany and Plant Sciences, Institute of Integrative Genome Biology, University of California, Riverside, CA 92521, USA

## Abstract

*Brassica parachinensis* is a popular leafy vegetable. It is able to accumulate high concentrations of cadmium (Cd), but the molecular mechanism of Cd accumulation is unknown. This study investigated the function and regulatory mechanism of the Cd-responsive metal ion transporter gene *BrpHMA2*. *BrpHMA2* was induced by Cd stress and specifically expressed in vascular tissues, and the protein was localized in the plasma membrane. Heterologous expression of *BrpHMA2* enhanced Cd accumulation and Cd sensitivity in transgenic *Arabidopsis* and yeast. After Cd stress, the transcription factors BrpNAC895 and BrpABI449, which may recognize the abscisic acid-responsive elements in the *BrpHMA2* promoter, were also differentially expressed. The transcriptional regulation of *BrpHMA2* was further investigated using the chromatin immunoprecipitation–quantitative PCR (ChIP–qPCR) assay, the electrophoretic mobility shift assay (EMSA), and luciferase (LUC) reporter activity analysis employing the transient expression system of *B. parachinensis* protoplasts and tobacco leaves and the *Escherichia coli* expression system. By binding to the promoter, BrpNAC895 induced the transcription of *BrpHMA2*. BrpABI449 might bind to the *BrpHMA2* promoter or interact with BrpNAC895 to interfere with the action of BrpNAC895. The findings suggest that BrpHMA2 is a membrane-based afflux-type Cd transporter involved in Cd^**2+**^ uptake and long-distance transport in plants. BrpNAC895 and BrpABI449, which function as the transcription activator and repressor, respectively, coregulate *BrpHMA2* expression.

## Introduction

Cadmium (Cd) is one of the major environmental pollutants and a potential hazard to worldwide agriculture. Excess Cd uptake in plants normally induces the accumulation of reactive oxygen species (ROS) in plants and has severe consequences, such as chromosome aberrations, protein inactivation, membrane damage, and and further leading to leaf chlorosis and root growth inhibition [1]. Furthermore, accumulation of Cd in crops enhances the risk of Cd poisoning in humans and animals [1]. *Brassica* species have been identified as Cd hyperaccumulators [2]. *Brassica parachinensis* L.H. Bailey (Chinese flowering cabbage) is a leafy vegetable widely consumed in China, Europe, and other regions of the world [3]. Thus, elucidating the molecular mechanisms of Cd accumulation in this plant is essential for developing effective strategies to control Cd accumulation in the plant’s edible parts.

Cd accumulation in plant tissues generally involves a three-step process: (i) absorption and accumulation of Cd in roots from the soil, (ii) translocation of Cd to the shoot via vascular tissue, and (iii) Cd storage in leaves [4]. Cd transporters are considered to play central roles in various physiological activities. The HMA (heavy metal ATPase), ZIP (zinc-regulated transporter protein), and Nramp (natural resistance-associated macrophage protein) families are among the transporter families that have been identified as being involved in these processes [5–7]. Our previous transcriptome analyses of *B. parachinensis* also showed that differentially expressed genes enriched in the gene ontogeny (GO) terms ‘transmembrane transport’ and ‘metal ion transport’ may be involved in response to Cd, including genes encoding members of some transporter families, such as the subfamily C of ATP-binding cassette proteins (ABCCs) and HMAs [8].

HMAs, which belong to the P1B subfamily of the P-type ATPase superfamily, have been extensively investigated in the model plant *Arabidopsis* as well as in some crop plants, and the main focus of these studies has been on their functions [9]. For example, eight members of HMAs have been identified in *Arabidopsis thaliana*, and among these, AtHMA1–AtHMA4 are thought to specifically transport divalent cations, such as Zn^2+^, Cd^2+^, Co^2+^, and Pb^2+^ [10]. AtHMA2 is generally regarded as a Zn^2+^-ATPase [11–15]. It contains a conserved short metal-binding domain in the N-terminus and a long metal binding domain in the C-terminal end; Zn^2+^-binding affinity was detected in both domains [13, 14], and Cd^2+^- and Cu^+^-binding affinity was detected in the N-terminal domain [13]. Some studies showed that AtHMA2 functioned as an efflux to drive the outward transport of metals from the cell cytoplasm and responsible for cytoplasmic Zn^2+^ homeostasis and Cd detoxification [14, 15]. Some researchers proposed that AtHMA2 together with AtHMA4 played key roles in the long-distance root-to-shoot transport of Zn^2+^ and Cd^2+^ by loading these ions into the xylem [10, 13]. Similar results were also reported in wheat TaHMA2 [16, 17]. However, it seems that OsHMA2 in rice has a different role. The enhanced sensitivity to Cd and tolerance to zinc (Zn) deprivation afforded by heterologous expression of *OsHMA2* in yeast cells suggest that OsHMA2 functions as a Cd influx transporter [18]. These studies showed that HMA2 and its subfamily members in different plants may function differently. There is a lack of thorough knowledge of the role of *BrpHMA2* in Cd hyperaccumulation in the leafy vegetable *B. parachinensis*. The function of *BrpHMA2* and the mechanisms that regulate its expression must be elucidated.

Previous studies have indicated that plants employ a universal and conserved approach to regulate the transcription of heavy metal uptake and tolerance genes. For example, in a bean (*Phaseolus vulgaris*), PvMTF-1 (metal response element-binding transcription factor 1), which could be induced by PvERF15 (ethylene response factor 15), may regulate the expression of the stress-related gene *PvSR2* and confer Cd tolerance to the plant [19]. In *Arabidopsis*, two basic helix–loop–helix (bHLH) transcription factors (TFs), FIT (Fer-like iron deficiency-induced transcription factor) and PYE (POPEYE), modulate iron (Fe) deficiency responses by regulating the expression of IRT1 and FRO2 [20], whereas the bHLH TFs IAA-leucine resistant 3 (ILR3) and bHLH104 can form heterodimers and bind to specific elements in the promoter of PYE to regulate PYE [21].

NAC (NAM, ATAF1, and CUC2) TFs are members of the most prominent TF families in plants. These TFs play essential roles in diverse biological processes, such as growth, development, senescence, and morphogenesis, and are widely involved in various signaling pathways in response to different phytohormones and multiple abiotic and biotic stresses [22, 23]. For example, NAC019, NAC055, and NAC072 negatively regulate drought stress-responsive signaling [24]. NAC096 is associated with drought stress. It could exert its function via a mechanism like that of basic leucine zipper protein (bZIP)-type TFs to bind specifically to abscisic acid (ABA)-responsive elements in the promoters of several drought stress-responsive genes [25]. This finding implies that NAC096 and bZIP-type TFs can sometimes regulate the same target genes [26]. Studies have also shown that the core DNA-binding sequences of NACRE (NAC-responsive element) and ABRE (ABA-responsive element) are PyCACG and PyACGTGG/TC (Py, pyrimidine), respectively [26].

In a previous study, we identified a few NAC and AREB (ABA-responsive element-binding protein) TFs triggered by Cd stress in *B. parachinensis* [8]. However, their functions remain unclear. To clarify the molecular mechanisms of Cd accumulation in *B. parachinensis*, the function of a Cd-responsive metal ion transporter gene *BrpHMA2* and the coregulation of *BrpHMA2* transcription by two TFs (BrpNAC895 and BrpABI449) were examined in this study. The findings reveal a precise regulatory mechanism in *B. parachinensis* in response to Cd stress.

## Results

### Expression pattern of *BrpHMA2*

We previously analyzed the Cd-induced mRNA transcriptome of *B. parachinensis* and found that several HMA homologs were substantially expressed under Cd stress [8]. We cloned one of the HMA2 homologs and constructed the phylogenetic tree of this *HMA2* homolog with other HMAs in *A. thaliana*, *Oryza sativa*, *Zea mays*, and Alfred stonecrop by the neighbor-joining method using MEGA5. The results revealed that the sequence of this *HMA2* homolog is closer to that of the *AtHMA2* gene (Supporting Information Figure S1), and thus it was named *BrpHMA2*.

**Figure 1:**
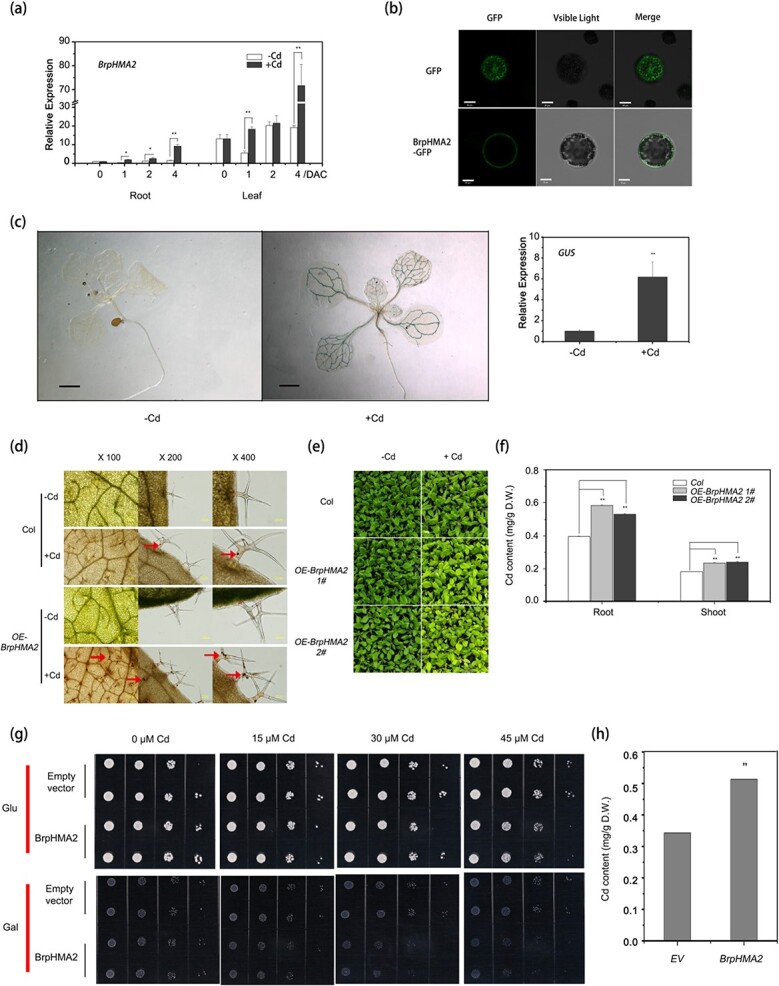
Expression pattern of *BrpHMA2* in *B. parachinensis* and the impact of *BrpHMA2* on Cd tolerance and accumulation in transgenic *Arabidopsis* and yeast cells. **a** Relative expression of *BrpHMA2* in *B parachinensis*. Fourteen-day-old hydroponic seedlings of *B. parachinensis* were treated with 50 μM Cd(NO_3_)_2_ for 1, 2 and 4 days; expression of *BrpHMA2* was then analyzed by RT–qPCR. DAC, days after Cd stress. **b** Subcellular localization of BrpHMA2. Protoplasts of *B. parachinensis* leaves with transient expression of BrpHMA2-GFP or GFP were observed under a confocal microscope. GFP fluorescence (left), bright-field (middle), and merged images (right) are shown. Scale bars: 20 μm. **c** Histochemical assays (left) and RT–qPCR analysis (right) of *pBrpHMA2::GUS* transgenic *Arabidopsis*. Analysis was performed on 9-day-old transgenic *Arabidopsis* seedlings treated for 2 days with or without Cd. Scale bar = 1 mm. **d** Cd localization in leaves of Col and *p35S::BrpHMA2* transgenic *Arabidopsis* (*OE-BrpHMA2*) seedlings. Arrows point to precipitates of Cd-dithizone. **e** Phenotype of transgenic plants and **f** Cd contents in *Arabidopsis* seedlings. Three-week-old hydroponic seedlings of *Arabidopsis* were exposed to 0 or 20 μM Cd(NO_3_)_2_ for 4 days for Cd precipitate observation and for 6 days for the phenotype observation and the Cd content analysis. **g** Growth of yeast cells transformed with empty vector or *BrpHMA2*. Cells were inoculated on SD plates containing different concentrations of Cd(NO_3_)_2_ in the presence of 2% glucose or 2% galactose and cultured for 3 days. **h** Cd content in yeast cells grown in liquid medium containing 2% galactose and 60 μM Cd(NO_3_)_2_ for 24 hours. EV, empty vector. Each experiment (biological repeat) used pooled RNA extracted from tissues collected from three independent plants in (**a**) and 25 seedlings in (**c**). Error bars represent the SD of three biological replicates. Asterisks indicate significant differences with respect to means of the control plants (Student’s *t*-test): ^*^P < .05; ^**^P < .01.

The transcript level of *BrpHMA2* in seedlings grown hydroponically was examined using reverse transcription–quantitative PCR (RT–qPCR) to investigate the expression pattern of *BrpHMA2* in *B. parachinensis.* According to the results, *BrpHMA2* was expressed at higher levels in leaves than in roots. Cd stress may increase *BrpHMA2* expression in leaves and roots, although *BrpHMA2* expression in leaves fluctuates owing to developmental regulation (Fig. 1a). The *GUS* gene was transformed and expressed in *Arabidopsis* using the promoter of *BrpHMA2* (*pBrpHMA2::GUS*) to corroborate the expression pattern, and histochemical assays were performed. Instant β-glucuronidase (GUS) staining for 0.5 hours showed that the GUS signal was visible in the vascular bundles of the leaves and roots of the plants treated with 50 μM Cd (NO_3_)_2_ for 2 days, but not in vascular bundles of seedlings that were not treated with Cd (Fig. 1c). Results from an examination of transcripts of the *GUS* gene in the reporter line were also consistent with these findings (Fig. 1c). This showed that *BrpHMA2* could be induced by Cd stress.

However, when the *pBrpHMA2::GUS* transgenic seedlings were subjected to GUS staining for 3 hours, a strong GUS signal could be observed in the vascular bundles of the cotyledons, true leaves, stems, petals, filaments, and the carpopodium of the seeds in young siliques. The blue GUS signal was particularly strong in the tissue junction regions where the vascular bundles were clustered (Figure S2). These results indicate that *BrpHMA2* may function primarily in transport in vascular tissues. The fluorescent signal of BrpHMA-GFP (GFP, green fluorescent protein) was detected at the plasma membrane by transient expression analysis in protoplasts of *B. parachinensis* leaf cells (Fig. 1b), indicating that BrpHMA2 is localized at the plasma membrane.

### Overexpression of *BrpHMA2* enhances cadmium accumulation in *Arabidopsis*

To investigate the physiological role of *BrpHMA2* in plants, transgenic *A. thaliana* lines expressing *BrpHMA2* (*OE-BrpHMA2*) were generated. The Cd distribution and accumulation in seedlings of Col and *BrpHMA2*-overexpressing lines (OE-1, OE-2) were investigated. Dithizone staining showed that Cd was mainly located in the epidermal hairs of the leaves in both Col and *OE-BrpHMA2* seedlings, but more Cd-dithizone precipitates were found in the *OE-BrpHMA2* lines (Fig. 1d). Although Cd stress inhibited the growth of both Col and transgenic plants, the extent of growth inhibition in the *OE-BrpHMA2* plants was stronger (based on leaf size and color) than in the Col plants after 6 days of Cd exposure (Fig. 1e). Moreover, Cd content assay revealed that the roots and shoots of the *OE-BrpHMA2* plants had considerably more Cd than those of the *Col* plants (Fig. 1f).

### Overexpression of *BrpHMA2* enhances cadmium accumulation in yeast

To further analyze the function of *BrpHMA2*, *BrpHMA2* fused with the galactose-inducible promoter was transformed into a Cd-hypersensitive yeast mutant, *△ycf* [18]. In the presence of the transcriptional inducer galactose, Cd^2+^ considerably inhibited the growth of yeast cells with heterologous expression of *BrpHMA2* compared with that of cells transformed with the empty vector (Fig. 1g). However, when gene expression was suppressed by the presence of glucose, no growth differences were detected between the cells transformed with *BrpHMA2* and those transformed with the empty vector. The Cd content in the heterologous transgenic cells grown in liquid medium was higher than that in the control cells (Fig. 1h). These results indicate that BrpHMA2 functions as an afflux-type Cd transporter.

### NACs and abscisic acid-responsive element-binding proteins are differentially expressed in *B. parachinensis* after cadmium stress

**Figure 2:**
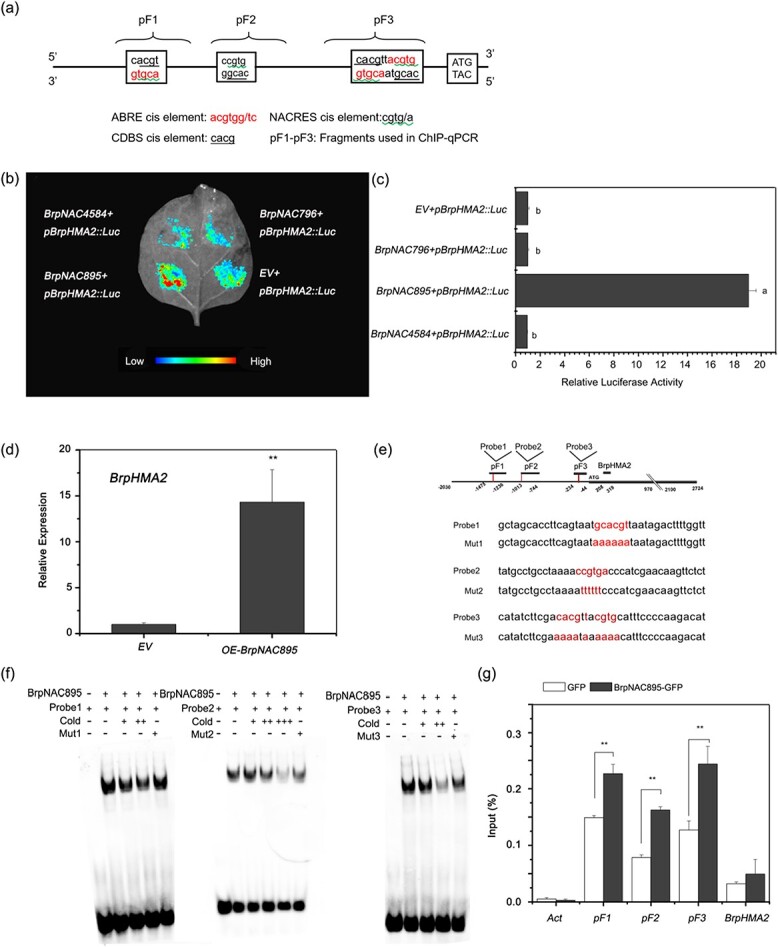
BrpNCA895 directly binds to the promoter of *BrpHMA2* and promotes *BrpHMA2* transcription. **a** Schematic diagram of the *cis* elements (ABRE, NACRES, CDBS) and the fragments used for ChIP–qPCR in the promoter of *BrpHMA2* (pF1–pF3). **b**, **c** Regulation of the expression of *pBrpHMA2::LUC* by BrpNAC895, BrpNAC4584 and BrpNAC796 analyzed in the transient expression system of (**b**) tobacco leaves and (**c**) *B. parachinensis* protoplasts. Different letters next to each bar indicate statistically significant differences as determined by one-way ANOVA followed by Tukey's multiple comparison test (*p* < .05). **d** Upregulated transcription of *BrpHMA2* detected in *B. parachinensis* protoplasts with transient expression of *BrpNAC895*. **e** Schematic diagram of probe positions in the *BrpHMA2* promoter and the probe sequences. **f** Binding of BrpNAC895-MBP to the promoter regions of *BrpHMA2* detected by EMSA. **g** Binding of BrpNAC895-GFP to *BrpHMA2* promoter regions detected by ChIP assay with an anti-GFP trap. *Actin* was used as a negative control. Asterisks indicate significant differences with respect to means of the control (Student’s *t*-test): ^**^P < .01.

To determine the TFs responsible for *BrpHMA2* expression in *B. parachinensis*, a *cis*-element analysis (PlantCARE) of 2000 bp of the *BrpHMA2* promoter was performed. In the promoter region, three ABRE *cis* elements (PyACGTGG/TC) were identified, all of which contain the G-box family core sequence ACGT (Fig. 2a). The NAC recognition site (NACRES) CGTG is likewise present in these ABREs. In the promoter of *BrpHMA2*, two additional NAC recognition motifs, CDBS (core DNA-binding sequence) and CACG, were found. Three ABREs (containing three NACRESs), four NACRESs, and four CDBS *cis* elements were found in the promoter of *BrpHMA2* (Fig. 2a). These findings suggest that certain transcription factors, such as NACs or AREBs, may control *BrpHMA2* in *B. parachinensis* via these *cis* elements. To confirm this deduction and identify the regulatory pathways involved in the response to Cd stress, the transcriptome of *B. parachinensis* as mentioned above was used to collect data for the *NAC* and *AREB* genes that showed differential expression following Cd stress [8]. Eighteen *NAC* genes and 11 *AREB* genes were selected to create a heat map, and three NAC TFs and three AREB TFs were identified as Cd-induced TFs (Figure S3). Their transcription levels were further analyzed by RT–qPCR. The results showed that the NAC TF genes *BraA03000895*, *BraA010004584*, and *BraA10002796* were upregulated in the roots of the plants exposed to Cd for 1 day (Figure S4a–c). After 4 days of Cd exposure, the AREB TF gene *BraA01000449* was induced in roots, and *BraA05001227*, *BraA01000449*, and *BraA01003678* were induced in leaves (Figure S4d–f). Similar to the findings for *BrpHMA2*, our results suggest that these TF genes may respond to Cd.

The coding sequences (CDSs) of the three *NAC* TFs and three *AREB* TFs listed above were cloned and submitted to the NCBI database. The last three or four numbers of each gene’s full name was used as the gene name. MEGA5 was used to create a phylogenetic tree of these *NAC* TF or *AREB* TF genes and *Arabidopsis* NAC or AREB genes using the neighbor-joining method. The results revealed that the *BrpNAC4584* and *BrpNAC895* sequences were closer to those of *Arabidopsis ANAC046* and *ANAC087*, respectively (Figure S5); in addition, the *BrpABI227* and *BrpABI678* sequences were closer to that of *AtABF4*, and the *BrpABI449* sequence was more comparable to that of *AtABF3* (Figure S6).

### BrpNAC895 binds to NAC-responsive element motifs in the promoter of *BrpHMA2* and upregulates *BrpHMA2* transcription

To determine whether the *NAC* TFs can regulate *BrpHMA2* expression, each *BrpNAC* driven by *35S* (*p35S*::*BrpNACs*) and the promoter of *BrpHMA2* fused to the firefly *LUC* (luciferase) gene (*pBrpHMA2::LUC*) were employed as effector and reporter, respectively, and cotransfected into tobacco leaves for a transient expression assay. The empty vector (pGreenII 0062SK) was used as a negative control of the effector. The cotransformation of *p35S*::*BrpNAC895* with *pBrpHMA2::LUC* resulted in stronger LUC fluorescence (Fig. 2b). The cotransformation of *p35S*::*BrpNAC895* and *pBrpHMA2::LUC* into protoplasts of *B. parachinensis* leaves could also result in a significant increase in the activity of the LUC reporter (Fig. 2c). Similarly, the transformation of *BrpNAC895* in *B. parachinensis* leaf protoplasts yielded a significant increase in the *BrpHMA2* transcription level (Fig. 2d). However, no positive regulation effects of the other two NAC TFs (BrpNAC796, BrpNAC4584) on the expression of LUC were detected (Fig. 2b and c). The results show that BrpNAC895 could promote the transcriptional activity of the *BrpHMA2* promoter.

Electrophoretic mobility shift assays (EMSAs) were conducted to investigate whether the BrpNAC895 protein directly binds to the promoter of *BrpHMA2*. Three probes containing NACRES and CBDS motifs on the *BrpHMA2* promoter were designed and used for the EMSA. The results revealed that the BrpNAC895-MBP (MBP, maltose binding protein) fusion protein could bind to the three probes *in vitro* (Fig. 2e and f). A chromatin immunoprecipitation (ChIP) assay was performed using an anti-GFP antibody to precipitate BrpNAC895-GFP fusion proteins expressed in *B. parachinensis* protoplasts, and three fragments (pF1, pF2, and pF3) covering the NACRES and CBDS motifs on the *BrpHMA2* promoter were designed and used for PCR. Moreover, there is only one base interval between the last two NACRES *cis* elements, so they were considered as one fragment (pF3) (Fig. 2a). Approximately 1.5- to 2-fold enrichment of fragments pF1, pF2, and pF3 was detected compared with those found in the control (Fig. 2e and g). The results demonstrate that BrpNAC895 can promote the expression of *BrpHMA2* by binding directly to the NACRES and CBDS motifs of its promoter*.*

### BrpNAC895 and BrpABI449 coregulate the expression of *BrpHMA2*

**Figure 3:**
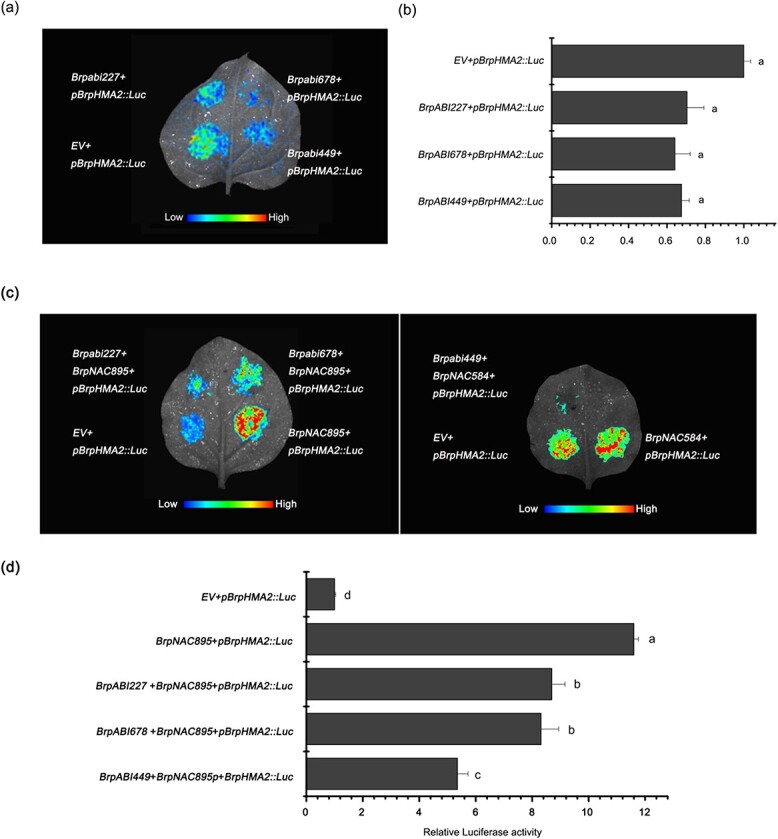
**a**, **b** Several *AREB* genes could reduce the BrpNAC895-activated transcription of *BrpHMA2*. Effect of *BrpABI449*, *BrpABI227*, and *BrpABI678* on the expression of *pBrpHMA2::LUC* when transiently expressed in (**a**) tobacco leaves and (**b**) protoplasts of *B. parachinensis*. **c**, **d** Coregulation of expression of *pBrpHMA2::LUC* by BrpNAC895 and several AREBs when transiently expressed in (**c**) tobacco leaves and (**d**) protoplasts of *B. parachinensis*. Different letters next to each bar in (**b**) and (**d**) indicate statistically significant differences as determined by one-way ANOVA followed by Tukey’s multiple comparison test (*P* < .05).

Transient expression assays were performed to determine the function of the selected AREB genes in regulating *BrpHMA2* transcription. The visualization of LUC activity revealed that these AREB genes could slightly downregulate the LUC activity of the reporter *pBrpHMA2::LUC*, even though the quantitative analysis indicated no significant difference between the transformations with each AREB gene or the empty vector (Fig. 3a and b). The complex relationship between NAC and AREB TFs has been confirmed mostly in *Arabidopsis* [25]. Three AREB TFs were cotransformed with *p35S::BrpNAC895* as the effector and *pBrpHMA2::LUC* as the reporter to assess the influence of AREB TFs on *BrpNAC895* function. The results showed that all three AREB TFs could reduce the BrpNAC895-activated transcription of *BrpHMA2*, and BrpABI449 exerted a stronger influence than the other two AREBs (Fig. 3c and d).

### BrpABI449 binds to the promoter of *BrpHMA2* directly

**Figure 4:**
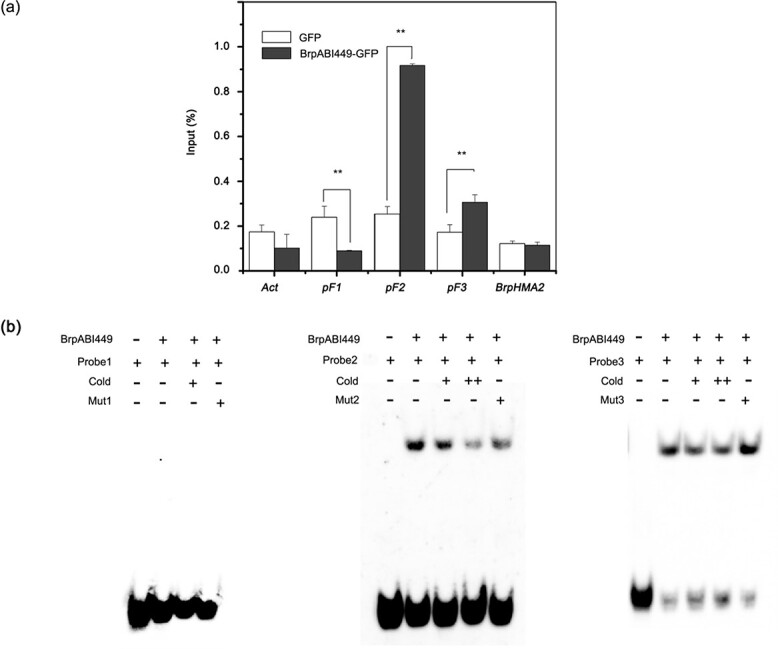
BrpABI449 directly binds to the *BrpHMA2* promoter. **a** Binding of BrpABI449-GFP to *BrpHMA2* promoter regions detected by ChIP assay with an anti-GFP trap. Actin was used as an unenriched control. **b** Binding of BrpABI449-MBP to the promoter regions of *BrpHMA2* detected by EMSA. Probes 1–3 and pF1–3 are the same as those shown in Fig. 2. Asterisks indicate significant differences with respect to means of the control (Student’s *t*-test): ^**^P < .01.

To investigate the mechanism of *BrpHMA2* coregulation by BrpNAC895 and BrpABI449, a ChIP assay was performed by expressing BrpABI449-GFP in *B. parachinensis* protoplasts to analyze the binding affinity of BrpABI449 with the promoter of *BrpHMA2*. A qPCR analysis revealed that the BrpABI449 protein was enriched with fragments containing pF2 and pF3 of the *BrpHMA2* promoter (Fig. 4a). We further performed an EMSA to confirm the binding of BrpABI449 to ABRE motifs in the promoter of *BrpHMA2*. The results proved that BrpABI449 could bind directly to the probes containing ABRE *cis* elements in the pF2 and pF3 regions of the *BrpHMA2* promoter (Fig. 4b).

### Two NAC-responsive element motifs in the promoter of *BrpHMA2* are required for BrpNAC895 transcriptional regulation

**Figure 5:**
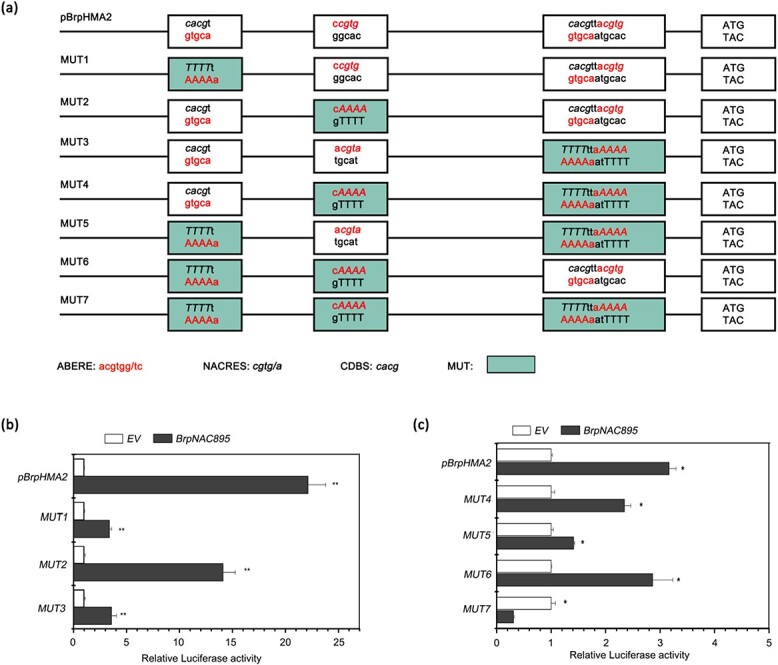
Roles of NACRE motifs in *BrpHMA2* transcription activated by BrpNAC895. **a** Schematic diagram of *pBrpHMA2* and its mutants *MUT1–7.***b**, **c** LUC activity detected in *B. parachinensis* protoplasts with transient expression of *35S::BrpNAC895* and *pBrpHMA2::LUC* or *pBrpHMA2 MUT::LUC.* EV, empty vector, employed as the control for *p35S::BrpNAC895*. Asterisks indicate significant differences with respect to means of the control (Student’s t-test): ^*^P < .05, ^**^P < .01.

The roles of BrpNAC895-binding loci in *BrpHAM2* transcriptional regulation were investigated by constructing a series of *BrpHMA2* promoter mutants (*MUT1*–*MUT7*) by changing CACG/CGTG in the NACRES or CBDS to AAAA (Fig. 5a). A dual LUC assay was performed using the effector *p35S::BrpNAC895* vector and the reporter vector was cotransformed into *B. parachinensis* protoplasts. Compared with *pBrpHMA2::LUC*, the cotransformation of *p35S::BrpNAC895* with *pMUT1::LUC* or *pMUT3::LUC* resulted in much reduced LUC activity, but the cotransformation of the *p35S::BrpNAC895* effector with *pMUT2::LUC* resulted in considerably higher LUC activity (Fig. 5b). Among the cotransformations of the promoter of *BrpHMA2* with two or more mutations, substantially weaker LUC activity could only be seen in the transformations with promoters mutated at both locus 1 and locus 3 (Fig. 5c). These findings indicate that the mutation in the first and third NACRES motifs reduced the BrpNAC895-activated transcription of *BrpHMA2*, and these two binding loci may play central roles in the BrpNAC895-activated transcription of *BrpHMA2*.

### BrpABI449 interacts with BrpNAC895 and decreases BrpNAC895 DNA-binding activity

**Figure 6:**
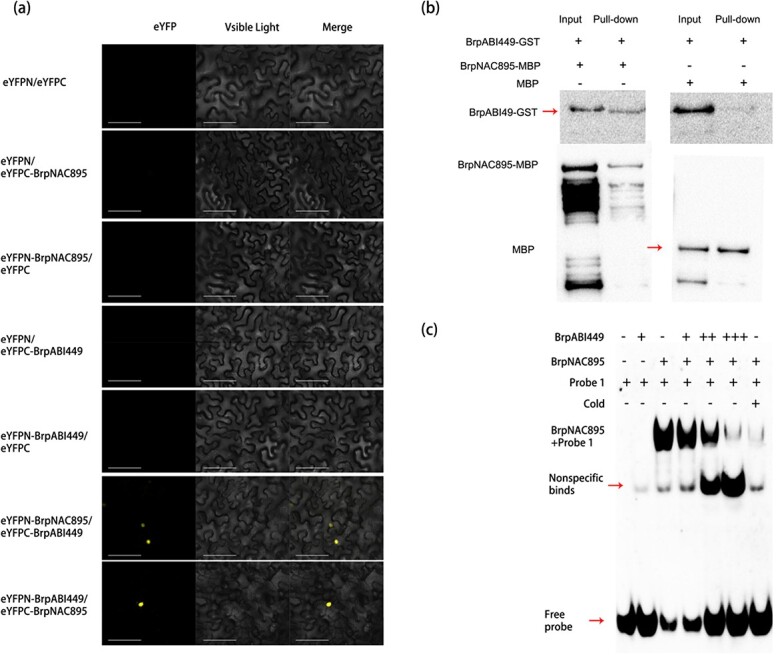
BrpABI449 interacts with BrpNAC895 in the nucleus and decreases BrpNAC895 DNA-binding ability. **a** BiFC assays in tobacco leaves. The C-terminal half of YFP and the N-terminal half of YFP were fused to BrpNAC895 or BrpABI449, respectively. Tobacco leaves were transfected with different combinations of constructs. Three days later, fluorescence was imaged using a confocal laser-scanning microscope. Scale bars = 100 μm. **b** Interactions of BrpNAC895 and BrpABI449 determined by pull-down assays. MBP-tagged BrpNAC895 and GST-tagged BrpABI449 purified from *E. coli* were used for pull-down. Pull-down assays were performed by incubating proteins with agarose for MBP protein affinity binding. The pull-down components were then analyzed by western blot with anti-GST or anti-MBP antibodies. MBP protein was used as a negative control in pull-down assays. **c** BrpABI449 decreased the DNA-binding activity of BrpNAC895 as determined by EMSA. MBP-tagged BrpNAC895 and MBP-tagged BrpABI449 were purified from *E. coli* cells and then incubated together for 6 hours at 4°C. EMSAs were performed by using these mixtures of proteins and Bio-tagged probes. Increasing numbers of + symbols represent increasing amounts of content added.

To elucidate the relationship between NAC and AREB TFs, a bimolecular fluorescence complementation (BiFC) approach was used. Full-length BrpNAC895 and BrpABI449 were fused to the C-terminal and N-terminal halves of enhanced yellow fluorescent protein (eYFP), respectively. The different fusion combinations were as follows: (i) eYFPN and eYPFC, (ii) eYFPN and eYPFC-BrpNAC895, (iii) eYFPN-BrpNAC895 and eYPFC, (iv) eYFPN and eYPFC-BrpABI449, (v) eYFPN-ABI449 and eYPFC, (vi) eYFPN-BrpNAC895 and eYPFC-BrpABI449, and (vii) eYFPN-BrpABI449 and eYPFC-BrpNAC895. No signal was detected in the transformations of combinations (i)–(v). When BrpNAC895 and BrpABI449 fused respectively with the C-terminal or N-terminal halves of eYFP were coexpressed in tobacco leaves, the BiFC signal could be detected in the nucleus. This indicates that BrpNAC895 could interact with BrpABI449 *in vivo* (Fig. 6a). To confirm this interaction *in vitro*, MBP-BrpNAC895 and BrpABI449-GST (GST, glutathione-*S*-transferase) fusion proteins were expressed in *Escherichia coli*, and protein pull-down was then performed by incubating dextrin beads with the incubated mixtures of MBP-BrpNAC895 or MBP (negative control) and BrpABI449-GST. The results demonstrated that BrpABI449 could be detected in the precipitates of MBP-BrpNAC895 (Fig. 6b) and indicate that BrpNAC895 can interact with BrpABI449.

The cotransfection of BrpABI449 with BrpNAC895 reduced the BrpNAC895-activated transcription of *BrpHMA2* (Fig. 3c and d); moreover, BrpABI449 could interact with BrpNAC895, which suggests that the interaction of BrpABI449 and BrpNAC895 inhibits the ability of BrpNAC895 to bind with the promoter of *BrpHMA2*. To confirm this speculation, we used an EMSA to analyze the binding ability of BrpNAC895 with the pF1 locus of the *BrpHMA2* promoter in the presence of the BrpABI449 protein. The results demonstrated that the binding of BrpNAC895 on the promoter fragment pF1 of *BrpHMA2* was reduced by the BrpABI449 protein (Fig. 6c). However, BrpABI449 could not bind to pF1 directly, as shown in Fig. 4c. These results demonstrate that the interaction between BrpABI449 and BrpNAC895 interferes with the binding of BrpNAC895 to the *BrpHMA2* promoter.

## Discussion

**Figure 7:**
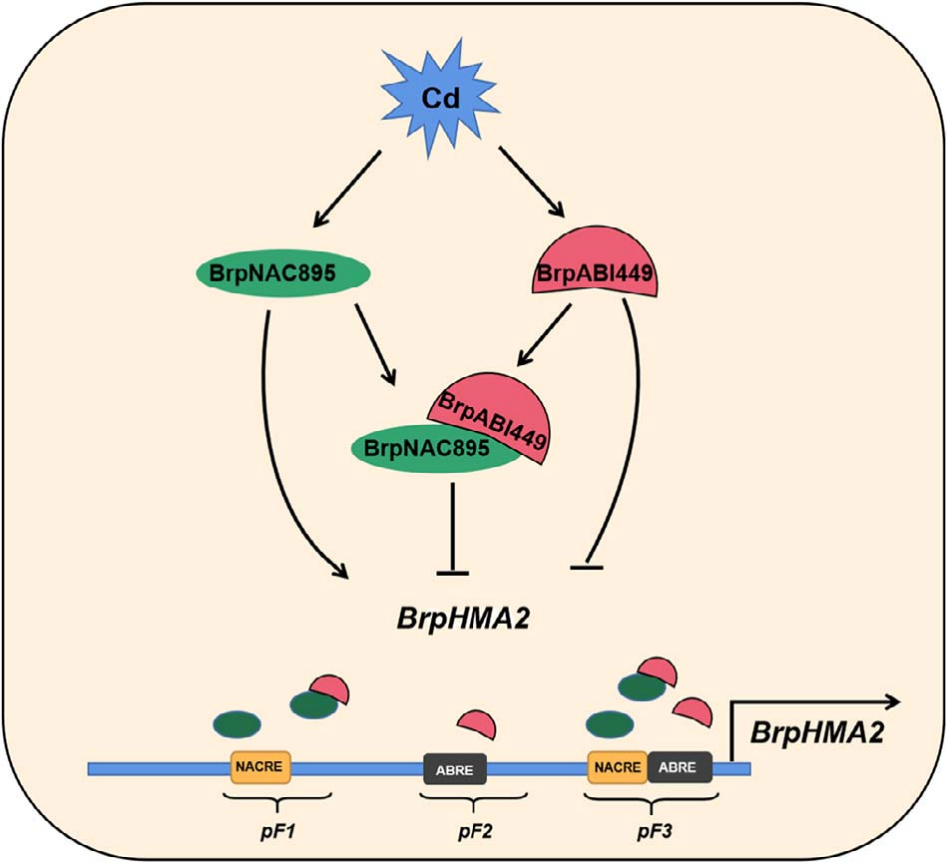
A proposed model for the transcriptional regulation of *BrpHMA2* by BrpNAC895 and BrpABI449 in *B. parachinensis*. Cd induces the expression of *BrpNAC895* and *BrpABI449.* BrpNAC895 functions as an activator to promote the transcription of *BrpHMA2* by binding to the loci pF1 and pF3 in its promoter. BrpABI449 could also bind to the promoter of *BrpHMA2* in the loci pF2 and pF3 or form a complex with BrpNAC895, and interfere with the binding action of BrpNAC895. BrpABI449 serves as a transcriptional repressor and coregulates the transcription of *BrpHMA2* with BrpNAC895.

*B. parachinensis* is a popular leafy crop; however, it may collect significant levels of heavy metals, particularly Cd, when grown on Cd-polluted substrate soil [2, 8, 27]. Transcriptomes of *B. parachinensis* under Cd stress were previously generated to elucidate the mechanisms underlying Cd accumulation [8]. We reveal that *BrpHMA2*, which is differently expressed in plants, is involved in Cd uptake and accumulation (Fig. 1). Furthermore, *BrpHMA2* expression is controlled by BrpNAC895 and BrpABI449, which operate as activators and inhibitors, respectively (Fig. 7).

### *BrpHMA2* functions in cadmium transport and is responsible for cadmium accumulation in plants

Our results reveal that *BrpHMA2* could be activated by Cd^2+^ (Fig. 1a), which is similar to the results found for *HMA2* in *Arabidopsis* [14]. Results suggest that *BrpHMA2* is involved in the Cd response of plants. *BrpHMA2* was also found to be expressed explicitly in the vascular tissues of roots, stems, leaves, flowers, siliques, and carpopodia, and its protein was localized in the plasma membrane (Fig. 1; Figure S2). These results are consistent with previous findings for HMA2 in *Arabidopsis* [15], OsHMA2 in rice, and TaHMA2 in wheat [16, 18]. The protein plasma membrane localization and the vascular-specific expression pattern of the genes (Fig. 1; Figure S2) revealed that HMA2 might function as a membrane transporter in long-distance transport in plants.

In recent years, some studies have investigated the function of HMA2. Most of these studies demonstrated that HMA2 is involved in Zn^2+^ and Cd^2+^ transmembrane transport and influences root-to-shoot Zn/Cd translocation. For example, HMA2 in *Arabidopsis* is thought to be involved in the outward transport of Zn^2+^ and Cd^2+^ from the cell cytoplasm, and HMA2 mutants are more sensitive to Cd stress and exhibit higher Zn or Cd accumulation than wild-type plants in the presence of high levels of Zn^2+^ or Cd^2+ 14,15^. The overexpression of *OsHMA2* in wheat, rice, and *Arabidopsis* improves root-to-shoot Zn/Cd translocation [16, 17]. In addition, the transformation of *TaHMA2* in yeast enhances the resistance of cells to Zn/Cd [16]. In rice, the suppression of *OsHMA2* decreases the Zn and Cd concentrations in leaves, increases the retention of Zn in roots and reduces the translocation of Cd and Zn from roots to shoots compared with the results obtained with wild-type plants [28]. According to the literature, HMA2 is responsible for Zn^2+^/Cd^2+^ efflux from cells, plays roles in Zn and Cd loading to the xylem, and participates in the root-to-shoot translocation of Zn/Cd.

However, Yamaji *et al*. [18] found that OsHMA2 is localized at the pericycle of the roots and in the phloem of enlarged and diffuse vascular bundles in the nodes. Their insertion lines of rice showed decreased concentrations of Zn and Cd in the upper nodes and reproductive organs. The study revealed that the heterologous expression of *OsHMA2* in yeast is associated with the influx transport of Zn and Cd. These researchers suggested that OsHMA2 in the nodes plays an important role in the preferential distribution of Zn and Cd through the phloem to the developing tissues [28]. Our results also revealed that, in the presence of Cd^2+^, transgenic *Arabidopsis* seedlings and yeast overexpressing *BrpHMA2* showed higher concentrations of Cd and enhanced Cd^2+^ sensitivity compared with the controls (Fig. 1). Thus, we propose that *BrpHMA2* functions in Cd^2+^ transport in the phloem tissue of vascular systems through influx into cells, and the efflux from phloem cells during long-distance transport may be performed by other transporters. The differential function of HMA2 from various plants might come from the tiny difference in amino acids in their function domains; this puzzle requires further investigation.

### Expression of *BrpHMA2* can be coregulated by BrpNAC895 and BrpABI449

In this study, we identified the NAC TF gene *BrpNAC895*, a homolog of *Arabidopsis ANAC087* (Figure S5), which could be induced by Cd^2+^ stress (Figure S4). We confirmed that *BrpNAC895* has a role in the response of *B. parachinensis* to Cd^2+^ stress by upregulating *BrpHMA2* expression through direct binding to the *BrpHMA2* promoter using EMSA, ChIP–qPCR, and the transient transformation method with *B. parachinensis* protoplasts (Fig. 2). Previous studies have demonstrated that *Arabidopsis ANAC087* is associated with plant programmed cell death (PCD). It functions along with the TF *ANAC046* to show partial redundancy in coregulating the expression of some PCD genes in the root columella, including *ZEN1*, *BFN1*, and *RNS3* [29]. Whether *ANAC087* could participate in regulating Cd transporters in plants has not been reported. Our findings on *BrpNAC895* show that this NAC TF has a novel role in upregulating *BrpHMA2* expression in response to Cd^2+^ stress.

We also identified the Cd-responsive AREB TF BrpABI449 (Figure S4), which is a homolog of *Arabidopsis* ABF3 (Figure S6) and can bind to the promoter of *BrpHMA2* (Fig. 4). ABF3 modulates the response to drought, salt, and other osmotic stresses as a master component in ABA signaling [30, 31]. This TF can also regulate the expression of multiple genes, such as the AGAMOUS-like MADS-box TF family gene *SOC1*, which is a floral integrator regulating flowering in response to drought [29], and the AREB TF ABI5, which is a core component in the ABA signaling pathway in the regulation of seed germination and early seedling growth during exposure to ABA and abiotic stresses [31, 32]. In general, ABF3 can form protein complexes with other TFs. For example, ABF3 forms homodimers or heterodimers with AREB1/AREB2 and acts cooperatively to regulate ABRE-dependent gene expression [30]. ABF3 forms a complex with NF-YC3 to promote the expression of the *SOC1* gene and thus accelerate flowering and drought-escape responses [29]; ABF3 interacts with NAC072 to regulate *RD29A* and *RD29B* expression in response to ABA [33]. Thus, complex formation might be the important functional mechanism by which ABF3 regulates gene transcription.

Using EMSAs and ChIP–qPCR assays, we found that BrpABI449 could directly bind to regions of the *BrpHMA2* promoter (Fig. 4). The interaction of BrpABI449 and BrpNAC895 was further confirmed by pull-down and BiFC assays (Fig. 6). The inhibition of BrpABI449 on the transcriptional regulatory role of BrpNAC895 was detected in the *B. parachinensis* protoplast transient system (Fig. 3). The inhibition by BrpABI449 of the transcriptional regulatory role of BrpNAC895 complex, likely interferes with BrpNAC895’s activity in the transcriptional activation of *BrpHMA2* in response to Cd stress. It has also been reported that Cd stress can induce a stress response via ABA signaling [34]. Our results showing that *BrpNAC895* and *BrpABI449* are upregulated by Cd stress also support this point.

The uptake or homeostatic regulation of heavy metals needs proper modulation to ensure plant health. Previous studies have shown that Cd stress induces the MYB TF gene *MYB49* in Arabidopsis. This TF may further positively regulate the downstream TF gene *bHLH38* and *bHLH101* by directly binding to their promoters, and activate iron-regulated transporter 1 (*IRT1*) to enhance Cd uptake [34]. In contrast, Cd stress upregulates the expression of *ABI5*. ABI5 interacts with MYB49, prevents its binding to the promoters of downstream genes, and functions as a negative regulator to control Cd uptake and accumulation [34]. Our present results also demonstrate a mechanism for controlling the expression of the heavy metal transporter gene *BrpHMA2* under Cd stress. We propose that Cd^2+^ induces the expression of *BrpNAC895* and *BrpABI449*, which might be mediated by ABA signaling. BrpNAC895 then promotes the transcription of *BrpHMA2* by binding directly to its promoter (Fig. 7). The activation of *BrpHMA2* enhances Cd^2+^ uptake and may induce cell damage. Negative regulation of *BrpHMA2* is then achieved by the upregulation of another AREB TF, BrpABI449, which interacts with BrpNAC895 and forms BrpNAC895-BrpABI449 protein complexes to inhibit the *BrpHMA2* transcription activated by BrpNAC895 (Fig. 7). BrpABI449 could also bind to the promoter of *BrpHMA2* directly to compete with BrpNAC895 in binding to the *BrpHMA2* promoter. This negative regulation may play a supplementary role in the uptake and transport of Cd.

### Application of *B. parachinensis* protoplasts

Many plant species of Brassicaceae, including *Arabidopsis*, turnip, and oilseed rape, can be genetically modified, but the creation of transgenic *B. parachinensis* remains difficult. Therefore, we overexpressed *BrpHMA2* in *Arabidopsis* to investigate the function of *BrpHMA2* and established a transient transformation system in *B. parachinensis* protoplasts to perform gene regulatory network analysis. Protoplasts have been widely used for subcellular protein localization and gene regulation analyses. In this study, the transient transformation of *B. parachinensis* protoplasts was demonstrated to be a powerful system for ChIP–qPCR analysis. Previous studies have applied a similar approach to *Populus trichocarpa* and *Brassica napus* [35–37]. Although the transient transformation system of *B. parachinensis* protoplasts was successfully used in this study of molecular mechanisms, the system cannot be easily used for phenotype and physiological analyses. The lack of BrpNAC895 and BrpABI449 transgenic *B. parachinensis* is a problem that severely limits research on this plant. New techniques, such as the transient reprogramming of plant traits via the transfection of RNA-based viral vectors using *Agrobacterium* and gene editing combined with fast-treated *Agrobacterium* coculture, may be useful approaches for comprehending gene function concerning physiology and for the further application of modifications of gene function to effectively control the accumulation of Cd in *B. parachinensis* [38, 39].

## Materials and methods

### Plant material and growth conditions

*A. thaliana* (wild-type Columbia-0), tobacco *(Nicotiana benthamiana*), and Chinese flowering cabbage (*B. parachinensis* L.H. Bailey, cultivar ‘YQ’) were used in this study.

The *B. parachinensis* cultivar ‘YQ’ was obtained from the Vegetable Research Institute of Guangdong Academy of Agricultural Sciences. Seeds were surface-sterilized, grown on 1/2 Murashige and Skoog (MS) medium for 7 days and then transferred to a simple hydroponic culture device with 1/2 Hoagland nutrient solution (pH 5.8–6.0) [8]. For Cd treatment, a final concentration of 50 μM Cd(NO_3_)_2_ was applied to the nutrient solution 7 days after transplantation. *Arabidopsis* seedlings were cultured in 1/2 Hoagland nutrient solution (pH 5.8–6.0) as described by Conn *et al*. [40]. The Cd treatments were conducted with 21-day-old *Arabidopsis* seedlings by adding Cd(NO_3_)_2_ to the nutrient solution at a final Cd^2+^ concentration of 20 μM. Tobacco plants were grown in a mix of soil, perlite, and vermiculite at a ratio of 1:1:1. All seedlings were grown at 22°C and 60% relative humidity with a 16-hour photoperiod (~100 mmol m^−2^ s^−1^).

### Analysis of the cadmium tolerance of yeast

The *Saccharomyces cerevisiae △ycf* mutant strain was used to investigate the function of *BrpHMA2* [16]. *BrpHMA2* was included in the pAG413 vector, and the resulting vector was transformed into *△ycf* mediated by polyethylene glycol as previously described by Ito *et al*. [41]. For the evaluation of Cd tolerance, cultures of the transformants were sequentially 10× diluted and 4 μl of each diluted culture was spotted on solidified SD medium containing 0, 15, 30 and 45 μM Cd(NO_3_)_2_ in the presence of 2% glucose, an expression suppressor, or 2% galactose, an expression inducer. The plates were incubated at 30°C for 3 days. For measurement of the Cd contents, yeast cells were grown in liquid medium containing 2% galactose with shaking at 200 rpm to induce heterologous gene expression. After 24 hours of culture, 60 μM Cd(NO_3_)_2_ was added to the medium, and the cells were incubated for an additional 24 hours and harvested by centrifugation.

### Analyses of metal element contents and cadmium localization

*Arabidopsis* seedlings treated with Cd for 6 days and yeast cells treated with Cd for 24 hours were harvested. The samples were washed thoroughly with deionized water, 10 mM EDTA, 10 mM CaCl_2_, and deionized water successively. The samples were then dried and digested with 65% HNO_3_ using a microwave digestion system (Ethos ONE, Milestone, Italy). The Cd contents were analyzed by inductively coupled plasma optical emission spectrometry (ICP-OES, Optima 7000DV, Perkin Elmer, USA). Three biological replicates were examined.

To analyze the distribution of Cd at the tissue level, intact fresh leaves of *Arabidopsis* were rinsed in deionized H_2_O and then infiltrated in a staining solution (15 mg of diphenylthiocarbazone in 30 ml of acetone, 10 ml of H_2_O, and 50 ml of glacial acetic acid) for 1 hour. After a brief rinse in deionized H_2_O, well-stained leaf samples were photographed under a light microscope (Eclipse E200, Nikon) to show the red-black Cd-dithizone precipitates [42].

### Gene cloning

The CDSs of *BrpHMA2*, *BrpNAC895*, *BrpNAC796*, *BrpNAC4578*, *BrpABI449*, *BrpABI227*, and *BrpABI678* were amplified from the cDNA of ‘YQ’ by PCR using PrimerStar (Takara). The PCR amplicons were cloned into the empty pEASY^®^-Blunt Zero Cloning vector (Transgen).

### β-Glucuronidase staining assay

The 2.0-kb promoter of *BrpHMA2* was amplified by PCR from the genomic DNA of YQ and cloned into the plp100 vector carrying the *GUS* reporter gene to generate the *BrpHMA2::GUS* structure. GUS staining of the transgenic line of *BrpHMA2::GUS* was performed as described by Ivanov *et al*. [43].

### Reverse transcription–quantitative PCR

Total RNA was extracted from the samples using the TRIzol reagent according to the manufacturer’s protocol (Takara). RT–qPCR was performed using TransStart^®^ Tip Green qPCR SuperMix (Transgen) with a Roche LightCycler 480 real-time PCR instrument. *Ubiquitin* and *Actin*7 were used as the internal control genes for *Arabidopsis* and *B. parachinensis*, respectively.

### Recombinant protein purification, electrophoretic mobility shift assays, and pull-down assays

The full-length coding sequence of *BrpNAC895* or *BrpABI449* was cloned into the pMAL c5x vector with an MBP tag. The constructs were transferred into *E. coli* BL21(DE3) for recombinant protein production, and the recombinant protein was induced with 1 mM isopropyl-beta-D-thiogalactopyranoside at 16°C for 12 hours in a shaking incubator at 100 rpm and purified using the pMAL Protein Fusion and Purification System (NEB). EMSA was performed with the EMSA/Gel-Shift kit (Beyotime, China) according to the users’ guide. For pull-down assays, BrpABI449 was fused to the tag of GST. The protein interactions were detected by western blot analysis using anti-MBP and anti-GST antibodies (Transgen).

### Gene expression analysis, dual-luciferase reporter assay and chromatin immunoprecipitation–quantitative PCR of *B. parachinensis* protoplasts

The CDS of NACs and bZIPs without a stop codon was cloned into the pGreenII 0062SK vector [44]. The promoter of *BrpHMA2* was fused upstream of the firefly luciferase gene (*LUC*) in the pGreenII0800-LUC vector, which contains 35S::RLuc (*Renilla* luciferase, REN) [44]. Protoplasts of *B. parachinensis* were prepared as described previously [36].

RNA extraction and gene expression analysis by RT–qPCR was performed as described above.

Dual LUC assays were performed using a Dual-Luciferase Reporter Assay Kit (Promega) as previously described [44]. The ability of TFs to bind to the promoter regions of *BrpHMA2* was indicated by the ratio of LUC to REN activity.

The CDS of *BrpNAC895* or *BrpABI449* without a stop codon was cloned into the pGreenII background for expression of the fusion protein with a GFP tag. As previously described, ChIP assays of *B. parachinensis* protoplasts were performed with slight modifications [42]. Briefly, ~50 μg of plasmid DNA of pGreenII-*BrpNAC895* or pGreenII-*BrpABI449* and 1 × 10^7^ protoplasts were used for each transfection. Chromatin was fragmented by sonication with a Covaris S220 (Thermo) to obtain fragments of ~500 bp. *Actin7* was used as the internal control. Transfection with the pGreenII 62-SK vector for the overexpression of GFP was used as a negative control. The primers used for ChIP–qPCR can be found in Supplemental Table S1. Three biological replicates of each transfection and three technical repeats of each biological replicate were included in the experiment.

### Transient expression assays in tobacco leaves

*BrpHMA2* fused to the firefly *LUC* gene (*BrpHMA2::LUC)* was constructed in pGreenII 0800-LUC as a reporter, and *BrpNACs* or other AREBs were constructed in the pGreenII 62-SK vector as effectors. pGreenII 62-SK and pGreenII 0800-LUC vectors were used for transient expression assays in tobacco leaves. The reporter and effector were transiently expressed in tobacco leaves mediated by *Agrobacterium* GV3101 (pSoup). *Agrobacterium*-infected plants were cultivated under low irradiance for 24 hours and then transferred to light for an additional 2 days [45]. The LUC signal in the transfected leaves was detected with a CCD camera (Vilber Newton 7.0).

### Subcellular localization and bimolecular fluorescence complementation assays

*BrpHMA2* was inserted into the 0062SK vector for the localization analysis of BrpHMA2-GFP. BrpNAC895 and BrpABI449 were fused to the N-terminus or C-terminus of YFP in the pGreen background, respectively. The constructs were then transformed or cotransformed into tobacco leaves by *Agrobacterium* strain GV3101 through injection-mediated transfection. Three days later, the fluorescence of GFP or YFP was imaged using a confocal laser-scanning microscope (Zeiss).

### Statistical analysis

All data comprised at least three biological replicates. Statistical analysis between the control and test groups was performed using Tukey’s test program. Significant differences were evaluated using one-way ANOVA. All analyses were performed using SPSS for Windows.

## Acknowledgements

This work was supported by the Natural Science Foundation of Guangdong Province (2020A1515010309), the Guangdong Innovation Research Team Fund (2014ZT05S078), and the Shenzhen Sustainable Development Science and Technology Project (KCXFZ20201221173211033).

## Author contributions

X.C. and Y.T. designed the research; S.L. performed the research; L.L. performed the LUC analyses; Y.D. conducted gene expression analyses; Y.B. contributed to the EMSA assays; C.S. contributed to the pull-down assays; S.H. and J.Z. contributed to protoplast isolation, L.S. and X.Y. performed the yeast cell analysis, and L.L. provided technical support. All authors read and approved the final manuscript.

## Data availability

All data supporting the study findings are available from the corresponding author upon reasonable request.

## Conflict of interest

The authors declare no conflicts of interest.

## Supplementary data

Supplementary data is available at *Horticulture Research* online.

## Supplementary Material

Web_Material_uhac044
