# Supplementary material for: BrpNAC895 and BrpABI449 coregulate the transcription of the afflux-type cadmium transporter BrpHMA2 in Brassica parachinensis
Source: Hortic Res. 2022 Feb 19;9:uhac044. doi: 10.1093/hr/uhac044 (PMC9045254; doi:10.1093/hr/uhac044)
Supplement: Web_Material_uhac044 [file web_material_uhac044.docx]

**Supporting Information Available**

**Figure S1.** Phylogenetic analysis of *HMA* genes.

**Figure S2.** GUS histochemical assays of *pBrpHMA2::GUS* transgenic Arabidopsis.

**Figure S3.** Expression profiles of the NAC family (a) and bZIP family (b) in response to Cd stress. Red and blue indicate up- and downregulation.

**Figure S4.** Expression of genes in the NAC and ABI families in *B.parachinensis* in response to Cd stress*.*

**Figure S5.** Phylogenetic analysis of *NAC* genes.

**Figure S6.** Phylogenetic analysis of *AREB* genes.

**Table S1.** Primer sequences used for cloning and qRT-PCR.


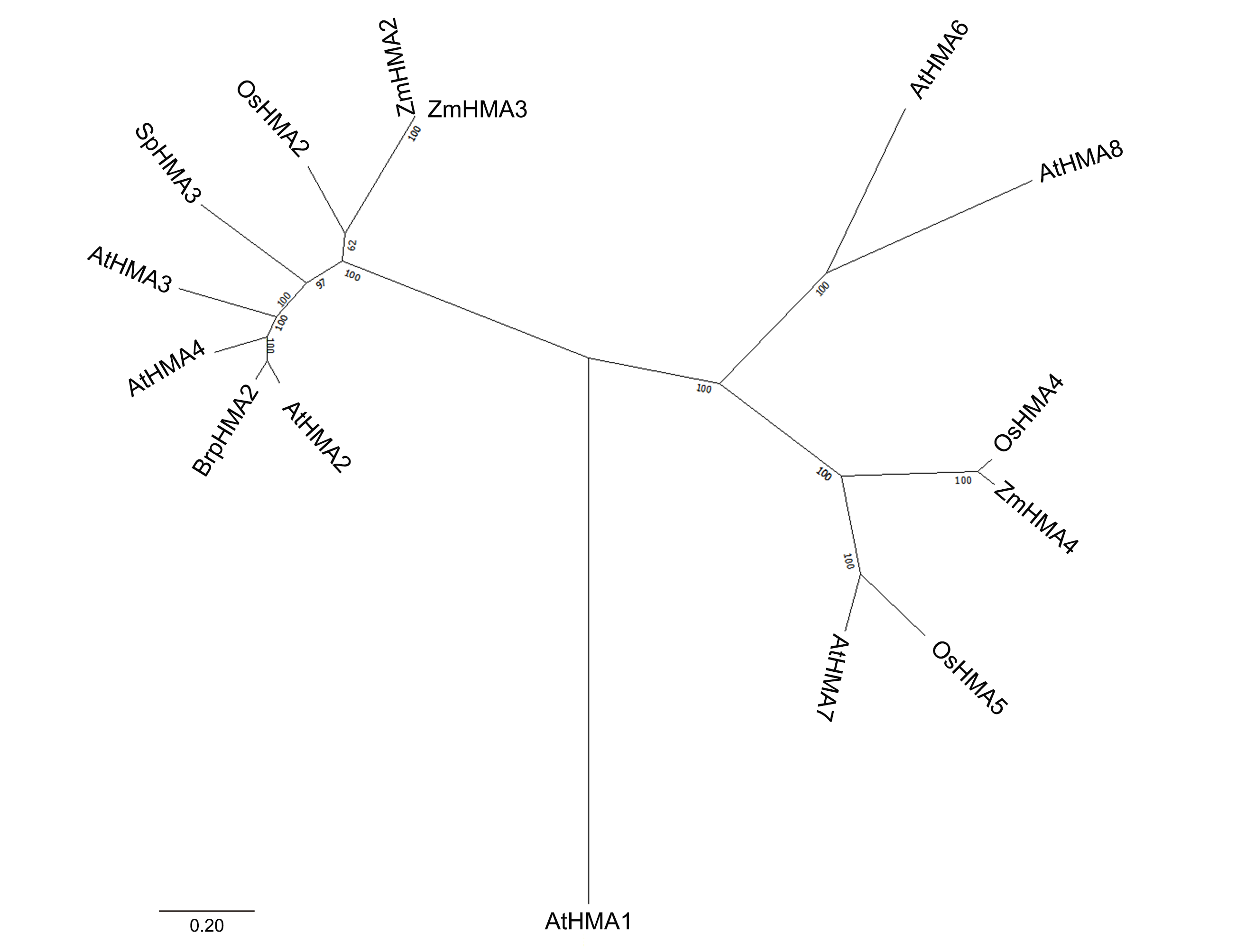


**Figure S1.** Phylogenetic analysis of *HMA* genes

Bootstrap values were obtained using 1000 replicates and are indicated before each embranchment of the phylogenetic tree. The tree was drawn to scale, with branch length corresponding to the number of amino acid substitutions per site. At = *Arabidopsis thaliana*, Os = *Oryza sativa*, Zm=*Zea mays*, Sp=*Sedum plumbizincicola，*Brp*= Brassica parachinensis.* HMA protein sequences from Arabidopsis and their identified orthologs in other species were retrieved from online databases: AtHMA1 (NM_119890), AtHMA2 (AY434728), AtHMA3 (AY055217), AtHMA4 (AF412407), AtHMA5 (Q9SH30), AtHMA6 (Q9SZC9), AtHMA7 (Q9S7J8), AtHMA8 (B9DFX7), OsHMA2 (HQ646362), OsHMA4 (Q6H7M3), ZmHMA2 (PWZ38732), ZmHMA4 (XP_008645432), SpHMA3 (ARK19360) and BrpHMA2 (MZ277875).


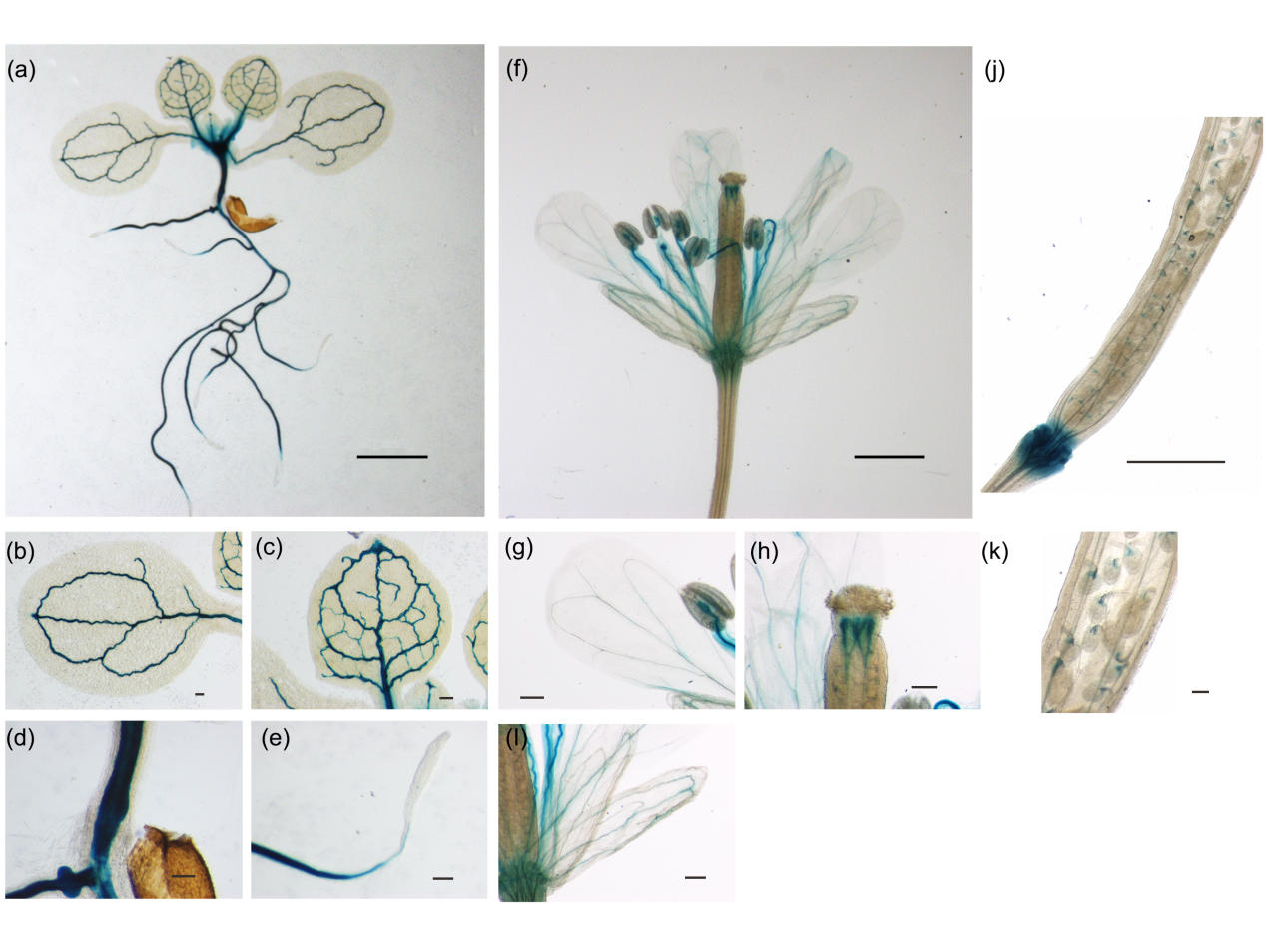


**Figure S2.** GUS histochemical assays of *pBrpHMA2::GUS* transgenic *Arabidopsis*.

GUS signals in 7-day-old seedlings: (a) the whole plant, (b) cotyledon, (c) true leafand (d) the junction of root and stem, (e) root. GUS signals in 40-day-old seedlings: [(f) to (i)] flowers and [ (j) and (k)] young siliques. Bars = 1 mm in (a), (f) and (j). 100 μm in (b) to (e), (g) to (i) and (k).


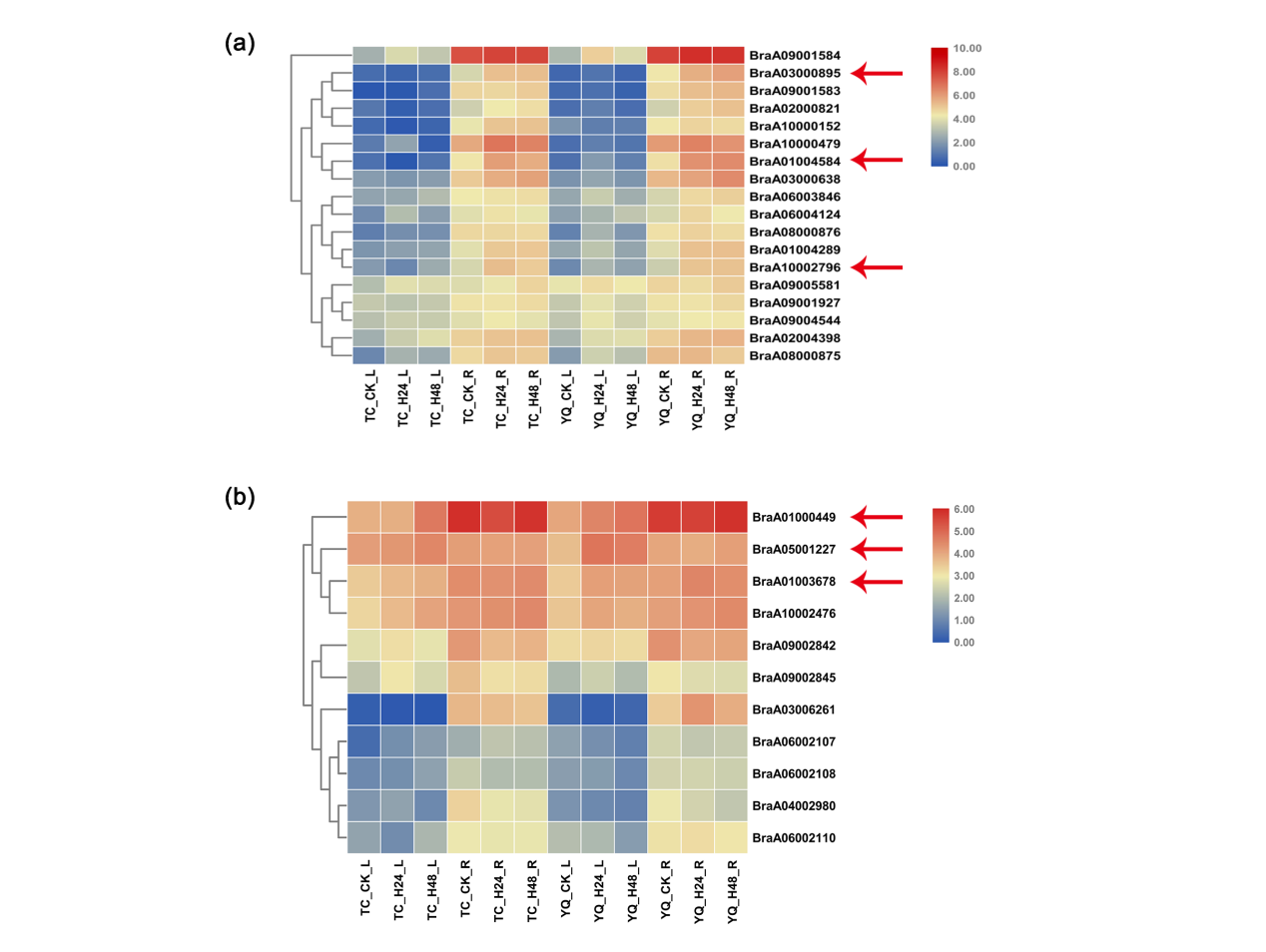


**Figure S3.** Expression profiles of the NAC family (a) and bZIP family (b) in response to Cd stress. Red and blue indicate up- and downregulation.


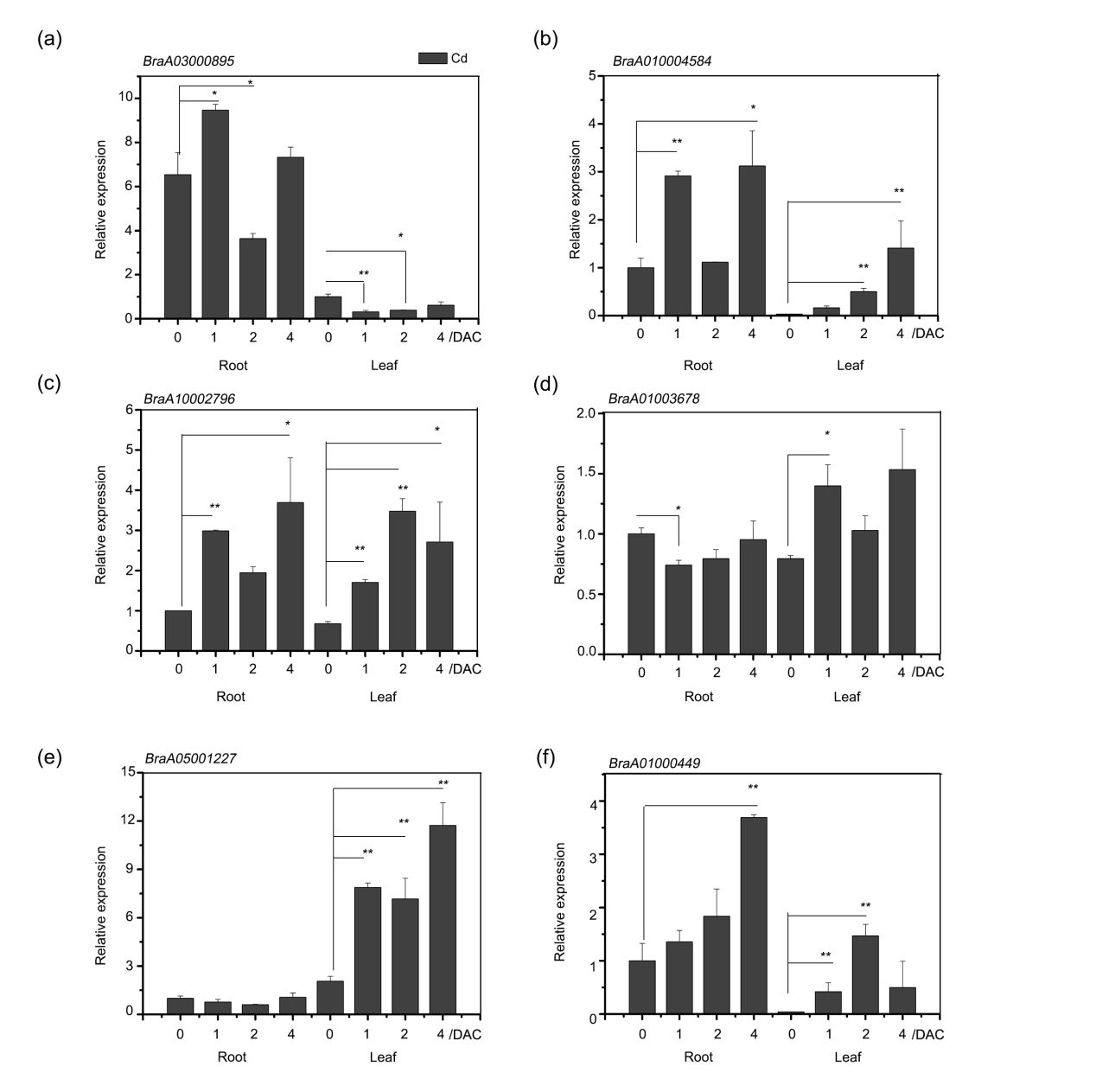


**Figure S4.** Expression of genes in the NAC and ABI families in *B.parachinensis* in response to Cd stress*.*

(a), (b) and (c) Expression of three NAC genes induced by Cd stress. (d), (e) and (f) Expression of three ABI genes induced by Cd stress. Error bars represent the SD of three biological replicates. DAC, days after Cd stress. Asterisks indicate significant differences with respect to means of the control plants (Student t test): * P < 0.05, ** P <0.01.


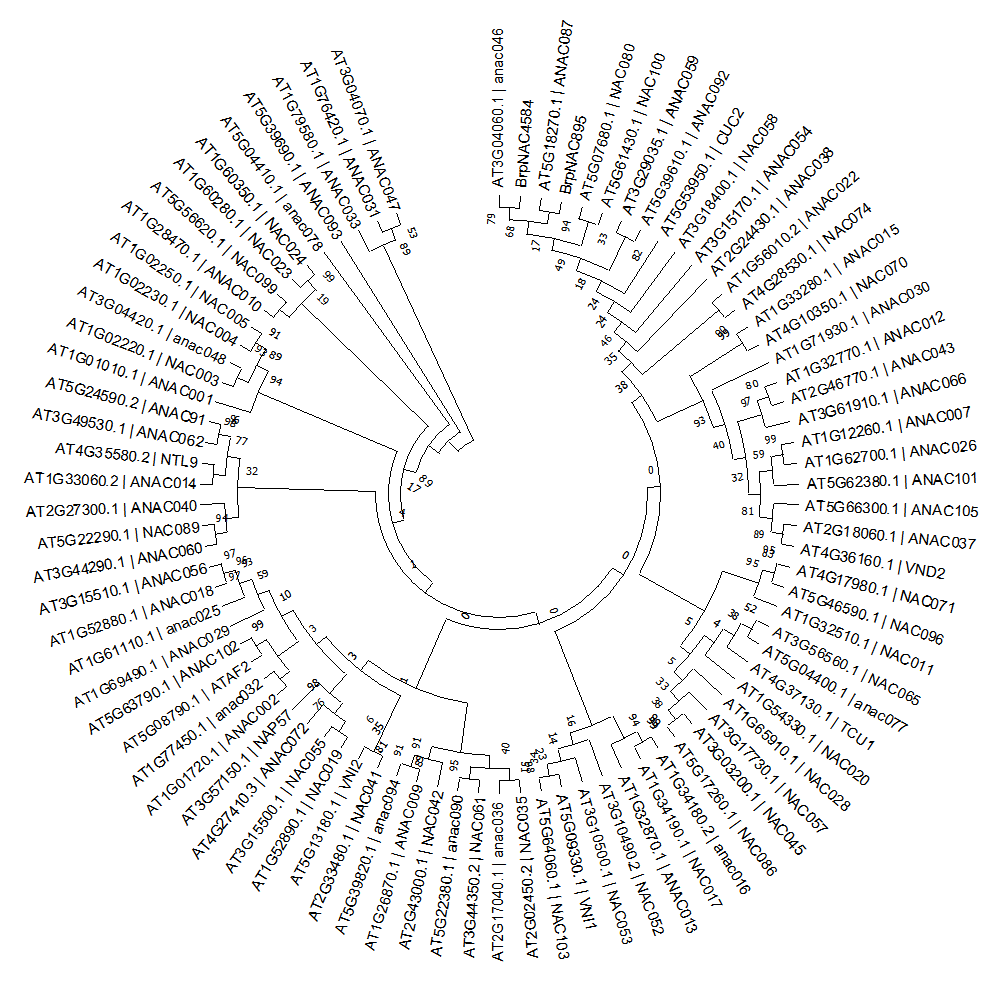


**Figure S5.** Phylogenetic analysis of *NAC* genes.

Bootstrap values were obtained using 1000 replicates and are indicated before each embranchment of the phylogenetic tree. The tree is drawn to scale, with branch length corresponding to the number of amino acid substitutions per site. At = *Arabidopsis thaliana*, Brp*= Brassica parachinensis. NAC* protein sequences from Arabidopsis were retrieved from TAIR.


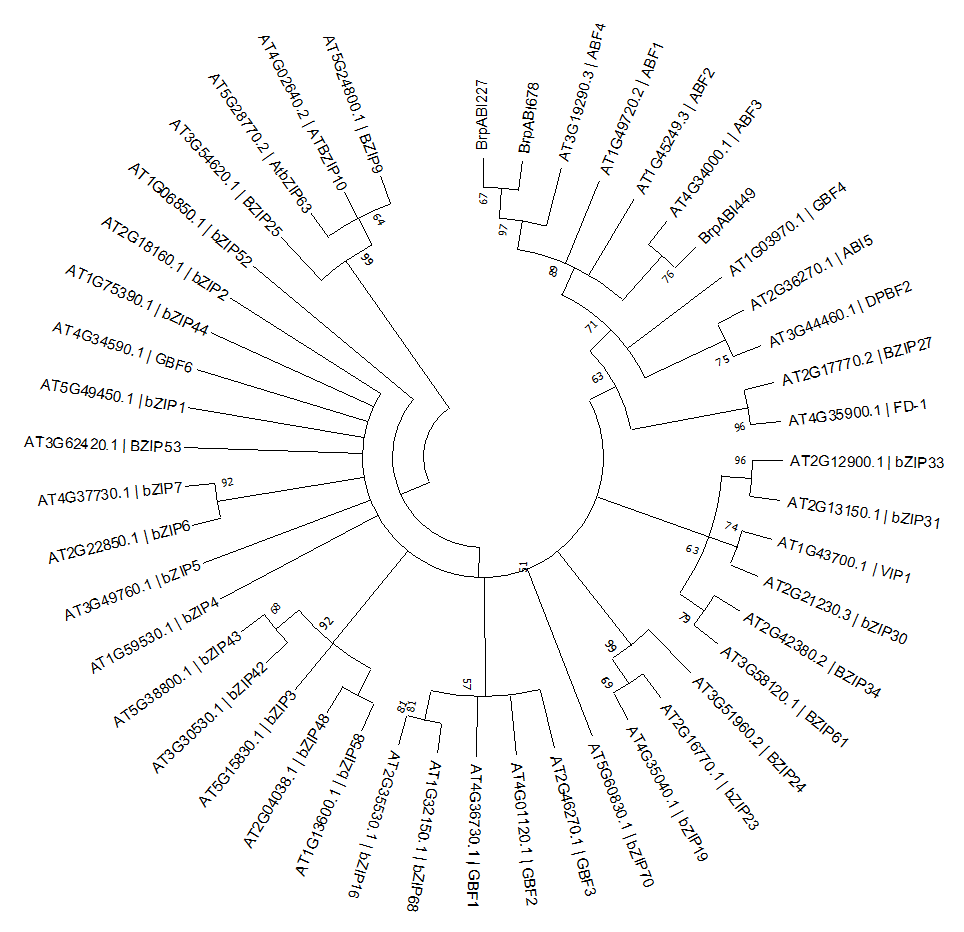


**Figure S6.** Phylogenetic analysis of *AREB* genes.

Bootstrap values were obtained using 1000 replicates and are indicated before each embranchment of the phylogenetic tree. The tree is drawn to scale, with branch length corresponding to the number of amino acid substitutions per site. At = *Arabidopsis thaliana*, Brp*= Brassica parachinensis. AREB* protein sequences from Arabidopsis were retrieved from TAIR.

**Table S1.** Primer sequences used for cloning and qRT-PCR.

| **Primer Name** | **Primer Sequences (5’-3’)** |
| --- | --- |
| **For cloning** |  |
| Brp449-0062-F(BamH 1) | tagaactagtggatccATGGGTTCTCAATTTAACTTCG |
| Brp449-0062-R(Hind111) | cggtatcgataagcttATAGCTTCTGCTCTTTGTTGAGCT |
| Brp678-0062-F(BamH 1) | tagaactagtggatccATGGGAACTCACGTCAATTTC |
| Brp678-0062-R(Hind111) | cggtatcgataagcttTACCATGGACCGGTTAGTGTCC |
| Brp227-0062-F(BamH 1) | tagaactagtggatccATGGGAACTCAGATCAATTTC |
| Brp227-0062-R(Hind111) | cggtatcgataagcttCACCATGGACCGGTTAATGTC |
| Brp895-0062-F(BamH 1) | tagaactagtggatccATGGCGGTTGTGGTAGAATCAG |
| Brp895-0062-R(Hind111) | cggtatcgataagcttCAGTAGTCCCATAAGATCC |
| Brp584-0062-F(BamH 1) | tagaactagtggatccATGGAGAGGAATCTCGTGGGATA |
| Brp584-0062-R(Hind111) | cggtatcgataagcttCAAGTTTTGACGCTTAGTG |
| Brp2796-0062-F(BamH 1) | tagaactagtggatccATGGTGGAAAAGCCAGGGTTT |
| Brp2796-0062-R(Hind111) | cggtatcgataagcttCTAACCAAGCAGAATAATCCAACC |
| pBrpHMA2-0800 Kpn1 F | tatagggcgaattgggtacctctctcaggggtaaaagg |
| pBrpHMA2-0800 Hind111r | attcgatatcaagctttcttatcgttcttggacgcc |
| BD-Brp895-EcoR1 F | CATATGGCCATGGAGGCCGAATTCATGGCGGTTGTGGTAGAATCAG |
| BD-Brp895-Pst1 R | TATGCTAGTTATGCGGCCGCTGCAGGCAGTAGTCCCATAAGATCC |
| BD-Brp449- EcoR1 F | CATATGGCCATGGAGGCCGAATTCATGGGTTCTCAATTTAACTTCG |
| BD-Brp449- Pst1 R | CTAGTTATGCGGCCGCTGCAGGATAGCTTCTGCTCTTTGTTGAGCT |
| AD-Brp895-EcoR1 F | ATGGCCATGGAGGCCAGTGAATTCATGGCGGTTGTGGTAGAATCAG |
| AD-Brp895-BamH1 R | ATCTGCAGCTCGAGCTCGATGGATCCCCAGTAGTCCCATAAGATCC |
| AD-Brp449- EcoR1 F | ATGGCCATGGAGGCCAGTGAATTCATGGGTTCTCAATTTAACTTCG |
| AD-Brp449- BamH1 R | CAGCTCGAGCTCGATGGATCCCATAGCTTCTGCTCTTTGTTGAGCT |
| pBrpHMA2 probe F1 | gctagcaccttcagtaatgcacgttaatagacttttggtt |
| pBrpHMA2 probe R1 | aaccaaaagtctattaacgtgcattactgaaggtgctagc |
| pBrpHMA2 Mutprobe F1 | gctagcaccttcagtaataaaaaataatagacttttggtt |
| pBrpHMA2 Mutprobe R1 | aaccaaaagtctattattttttattactgaaggtgctagc |
| pBrpHMA2 probe F2 | tatgcctgcctaaaaccgtgacccatcgaacaagttctct |
| pBrpHMA2 probe R2 | agagaacttgttcgatgggtcacggttttaggcaggcata |
| pBrpHMA2 Mutprobe F2 | tatgcctgcctaaaaTTTTTTcccatcgaacaagttctct |
| pBrpHMA2 Mutprobe R2 | agagaacttgttcgatgggAAAAAAttttaggcaggcata |
| pBrpHMA2 probe F3 | catatcttcgacacgttacgtgcatttccccaagacat |
| pBrpHMA2 probe R3 | atgtcttggggaaatgcacgtaacgtgtcgaagatatg |
| pBrpHMA2 Mutprobe F3 | catatcttcgaaaaatAAAAAAcatttccccaagacat |
| pBrpHMA2 Mutprobe R3 | atgtcttggggaaatgTTTTTTatttttcgaagatatg |
| BrpNAC895mby c5x-F | CCATGGGCGGCCGCATGGCGGTTGTGGTAGAAT |
| BrpNAC895mby c5x-R | ATTACCTGCAGGGAATTCTCAGTAGTCCCATAAGA |
| BrpABI449mby c5x-F | TCCATGGGCGGCCGCATGGGTTCTCAATTTAACTT |
| BrpABI449mby c5x-R | CTGCAGGGAATTCCTATAGCTTCTGCTCTTTGTTG |
| pBrapHAM2 FMUT3 | gctagcaccttcagtaatgAAAAttaatag |
| pBrapHAM2 rMUT3 | aTTTTcattactgaaggtgctagctgtgc |
| pBrapHAM2 rMUT2 | gggtTTTTgttttaggcaggca |
| pBrapHAM2 FMUT2 | gcctaaaacAAAAacccatcgaac |
| pBraHMA2 rMUT1 | ttcgacacgtaaaaaacatttccccaagacatta |
| pBraHMA2 FMUT1 | ttggggaaatgttttttacgtgtcgaagatatgt |
| **For Real-time PCR** |  |
| BrpHMA2 Fchipq2 | GAAGCATTCCCTGTGCCTAAA |
| BrpHMA2 Rchipq2 | CTTCCACCAGCTTAGCCATCT |
| pBrpHMA2 pN3 | TCGCCAAGTGTAGAGTGAACC |
| pBrpHMA2 pN3 | CTGCCAAAGAACGAAACCAAA |
| chip pHMA2 pN1F | CGCCAGTAAAGGACATAAAGC |
| chip pHMA2 pN1R | AACGTGCATTACTGAAGGTGC |
| chip pHMA2 pN2F | TATGCCTGCCTAAAACCGTGAC |
| chip pHMA2 pN2R | CATACTGAGACTGAAGAAACG |
| BrpABI449QF | GTTGACGACGCAAAGCAACCA |
| BrpABI449QR | ACCCAAAGTCCTTCCCAACCC |
| BrpABI227QF | CACAGCAGATGGGTCAGGTCA |
| BrpABI227QR | TGCTGTTGTGGCTGTTGTTGA |
| BrpNAC895QF1 | CAACCCTCCAACCTACGACAT |
| BrpNAC895QR1 | CATTACCGGACCCGAAGACAC |
| BrpNAC4584QF1 | TACCCATCAGCCCAAACCCTC |
| BrpNAC4584QR1 | ACCAGTCCCATAACCAGAACC |
| BrpABI3678QF | AGCAGCAGCGGATGCCTCAAA |
| BrpABI3678QR | CTCCTCCAAAGCTACCCAATC |
| BrpNAC796QF1 | TCGTGGGTTTCTATCTCCGTCAG |
| BrpNAC796QR1 | AAGGCGTCGTCCTGCTCTGT |
| gus QF2 | CGAACACCTGGGTGGACGATAT |
| gus QR2 | AAGTCCCGCTAGTGCCTTGTCC |
